# Δ40p53 is involved in the inactivation of autophagy and contributes to inhibition of cell death in HCT116-Δ40p53 cells

**DOI:** 10.18632/oncotarget.14460

**Published:** 2017-01-03

**Authors:** Yunjin Zang, Ying Shi, Kai Liu, Luxin Qiao, Xianghua Guo, Dexi Chen

**Affiliations:** ^1^ Beijing Institute of Hepatology, Beijing 100069, China; ^2^ Capital Medical University affiliated Beijing You An Hospital, Beijing 100069, China; ^3^ Organ Transplantation Center, The Affiliated Hospital of Qingdao University, Shandong Province, 266003, China

**Keywords:** Δ40p53, wild-type p53, autophagy, cell death

## Abstract

Δ40p53 is an isoform of wild-type p53 (wtp53). Here, we assessed whether Δ40p53 has the same functions as wild-type p53 in the regulation of cell death and autophagy. First, we used HCT116 (p53+/+) and H1299 (p53-free) cells to produce two cell lines (HCT116-Δ40p53 and H1299-Δ40p53) that express exogenous Δ40p53 but not wtp53. By using these cell lines, we determined that Δ40p53 inhibited starvation-induced autophagy, as does wtp53. This inhibition arises from both Δ40p53 and wtp53 having 3′-5′ exonuclease activity, which reduces the levels of double-stranded RNA (dsRNA) and then inhibits PKR/eIF2α-induced autophagy in cells exposed to starvation. Like wtp53, the translocation of Δ40p53 to the nucleus increased in cells in response to Methyl methane sulfonate (MMS) treatment-induced DNA damage. Previous studies have shown that nuclear wtp53 can induce DRAM expression and DRAM-induced autophagy in cells in response to DNA damage, thereby contributing to apoptotic cell death as DRAM-induced autophagy is a pro-apoptotic factor. Here, nuclear Δ40p53 did not individually induce DRAM-induced autophagy and cell death in response to DNA damage. However, nuclear Δ40p53 inhibited wtp53-induced DRAM expression and cell death. Thus, Δ40p53 and wtp53 have 3′-5′ exonuclease activity and inhibit starvation-induced autophagy in the cytoplasm; however, nuclear Δ40p53 inhibits wtp53-induced cell death by impairing the transactivation activity of wtp53. Because wtp53 inhibits tumor and viral infection by inhibiting autophagy and promoting degradation of viral dsRNA, it is reasonable to believe that Δ40p53 has the similar functions. A deeper study of these functions of Δ40p53 is needed in the future.

## INTRODUCTION

Impairments in cell death pathways caused by mutation or loss of cell death regulators usually contribute to tumor development. In particular, wild-type p53 (wtp53), a well-known tumor suppressor, plays a key role in the maintenance of genomic stability through regulating DNA repair, cell-cycle checkpoints, autophagy and apoptosis [1, 2]. The ability of wtp53 to induce cell cycle arrest or cell death is taken advantage of in treating tumors. The function of wtp53 is determined by its location. The translocation of wtp53 to mitochondria induces early apoptosis [3]. When wtp53 is located in the nucleus, it transactivates several cell cycle arrest- or pro-apoptosis-related genes, which contributes to cell cycle arrest or apoptotic cell death [4]. DRAM was the first target gene identified to be induced by nuclear wtp53, connecting wtp53 with autophagy development [5]. DRAM-mediated autophagy is a pro-apoptotic factor; thus, wtp53 can induce apoptosis by the induction of autophagy development (termed autophagic apoptosis) [5].

Recent studies have also shown that when wtp53 is retained in the cytoplasm, it inhibits autophagy induced by starvation, rapamycin, lithium, or endoplasmic reticulum damage [2]. The 3′-5′ exonuclease activity of wtp53 might contribute to wtp53-inhibited autophagy, since the 3′-5′ exonuclease activity of wtp53 degrades double-stranded RNA (dsRNA), which results in inactivation of the PKR/eIF2α pathway. This, in turn, contributes to inhibition of the expression of some autophagy-related genes: e.g., Atg5 and Atg12 [6].

Δ40p53 is an isoform of wtp53 that can be generated by either alternative splicing of intron 2 or initiation of translation [7]. The effects of Δ40p53 on the function of wtp53 are contradictory. Δ40p53 has been shown to inhibit both wtp53 transcriptional activity and wtp53-induced apoptosis [7]. Δ40p53 has also been reported to activate the transcription of wtp53 target genes through a second transactivation domain located between amino acids 43 and 63 [8, 9]. Generally, the Δ40p53 level is low in most normal cells [10]; however, Δ40p53 is significantly upregulated in some tumor tissue [11]. Until now, studies investigating the functions of Δ40p53 have been rare.

In this study, we studied the role of Δ40p53 in the regulation of cell death and autophagy in depth. Methyl methane sulfonate (MMS) treatment is a common way to induce DNA damage and apoptotic cell death [12]. MMS-induced DNA damage also contributes to cell death by promoting wtp53-induced DRAM expression and the development of DRAM-mediated autophagic apoptosis [13]. The depletion of serum from medium contributes to starvation, which is a common method of inducing autophagy. Here, we assessed the effect of Δ40p53 on autophagy and cell death in cells treated with serum free-media and MMS.

## RESULTS

### MMS-induced DNA damage does not change the total level of Δ40p53 but induces the nuclear translocation of Δ40p53

**Figure 1:**
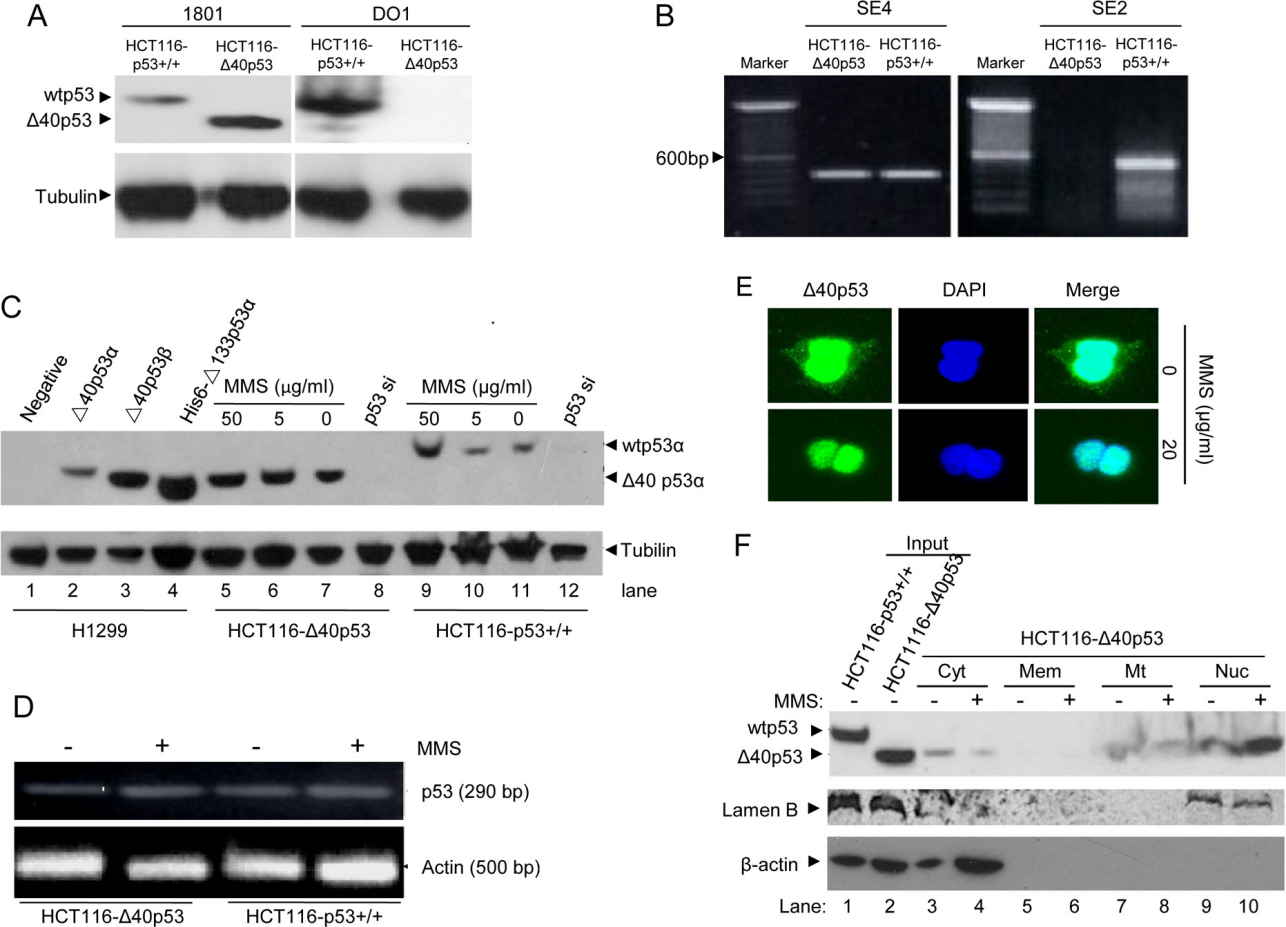
MMS treatment induces nuclear translocation of Δ40p53 but has no effect on its expression (**A**) Immunoblot detection of wtp53 and Δ40p53 in HCT116-p53+/+ and HCT116-Δ40p53 cells by using the anti-p53 antibodies 1801 and DO1. (**B**) qRT-PCR detection of the wtp53 and Δ40p53 mRNA in HCT116-p53+/+ and HCT116-Δ40p53 cells. SE2 and SE4 indicate amplification from exon 2 and 4 by using the p53SE2/p53-AS12 and p53SE4/p53-AS12 primer pairs, respectively. (**C**) Immunoblot detection of wtp53 and Δ40p53 in HCT116-p53+/+ and HCT116-Δ40p53 cells with 5 or 50 μg/ml MMS treatment. H1299 cells with or without transfection with Δ40p53 were used as a control. p53-directed siRNA (p53 si) was used to knockdown wtp53 and Δ40p53. (**D**) Detection of the wtp53 and Δ40p53 mRNA levels in HCT116-p53+/+ and HCT116-Δ40p53 cells with or without MMS treatment. (**E**) Immunofluorescence detection of the location of Δ40p53 in HCT116-Δ40p53 cells with or without MMS treatment. (**F**) Immunoblot detection of the level of wtp53 and Δ40p53 in isolated cytoplasm (Cyt), membrane (Mem), mitochondria (Mt) and nucleus (Nuc) fractions from HCT116-Δ40p53 cells with or without MMS treatment. The total protein from HCT116-p53+/+ and HCT116-Δ40p53 cells was used as the input control.

The antibodies 1801 and DO1 recognize the amino acid sequences of wtp53 from 46 to 55 and from 20 to 25, respectively. Thus, antibody 1801 has the ability to detect the expression of both Δ40p53 and wtp53, whereas antibody DO1 only detects wtp53. The immunoblot assay showed that Δ40p53 and wtp53 were detected in HCT116-Δ40p53 and HCT116-p53+/+ cells, respectively (Figure 1A). Moreover, Δ40p53 and wtp53 mRNA were detected by qRT-PCR in HCT116-Δ40p53 and HCT116-p53+/+ cells. The results of qRT-PCR showed that Δ40p53 mRNA but not wtp53 mRNA was present in HCT116-Δ40p53 cells, suggesting that wtp53 is depleted from HCT116-Δ40p53 cells and that these cells express only Δ40p53 mRNA (Figure 1B). Thus, HCT116-Δ40p53 is a wtp53-free cell line and expresses only exogenous Δ40p53.

Then, we used HCT116-p53+/+ and HCT116-Δ40p53 cells to detect the effect of MMS-induced DNA damage on the expression and sublocalization of Δ40p53. First, using different concentrations of MMS to treat HCT116-p53+/+ and HCT116-Δ40p53 cells, we determined that 50 μg/ml MMS induced more wtp53 expression in HCT116-p53+/+ cells than treatment with 5 μg/ml MMS, but MMS did not affect the level of Δ40p53 in HCT116-Δ40p53 cells (Figure 1C). Although the protein level of wtp53 significantly changed, neither p53 nor Δ40p53 mRNA was affected by MMS treatment (Figure 1D). Next, the immunofluorescence assay showed that Δ40p53 localized to the cytoplasm and nucleus under the control conditions, but MMS treatment resulted in the nuclear translocation of Δ40p53 in HCT116-Δ40p53 cells (Figure 1E). Last, through isolating the fragments of cytoplasm, membrane, mitochondria and nucleus of HCT116-Δ40p53 cells, we identified that Δ40p53 was present in the cytoplasm, mitochondria and nucleus, and MMS treatment mainly promoted the translocation of Δ40p53 from the cytoplasm to the nucleus (Figure 1F). Taking our findings together, we showed that although MMS treatment increases only wtp53 levels but not Δ40p53 levels, it does affect the sublocalization of Δ40p53, as shown by its promoting the translocation of Δ40p53 from the cytoplasm to the nucleus.

### Cytoplasmic Δ40p53 is an autophagy inhibitor, but nuclear Δ40p53 cannot induce cell death

**Figure 2:**
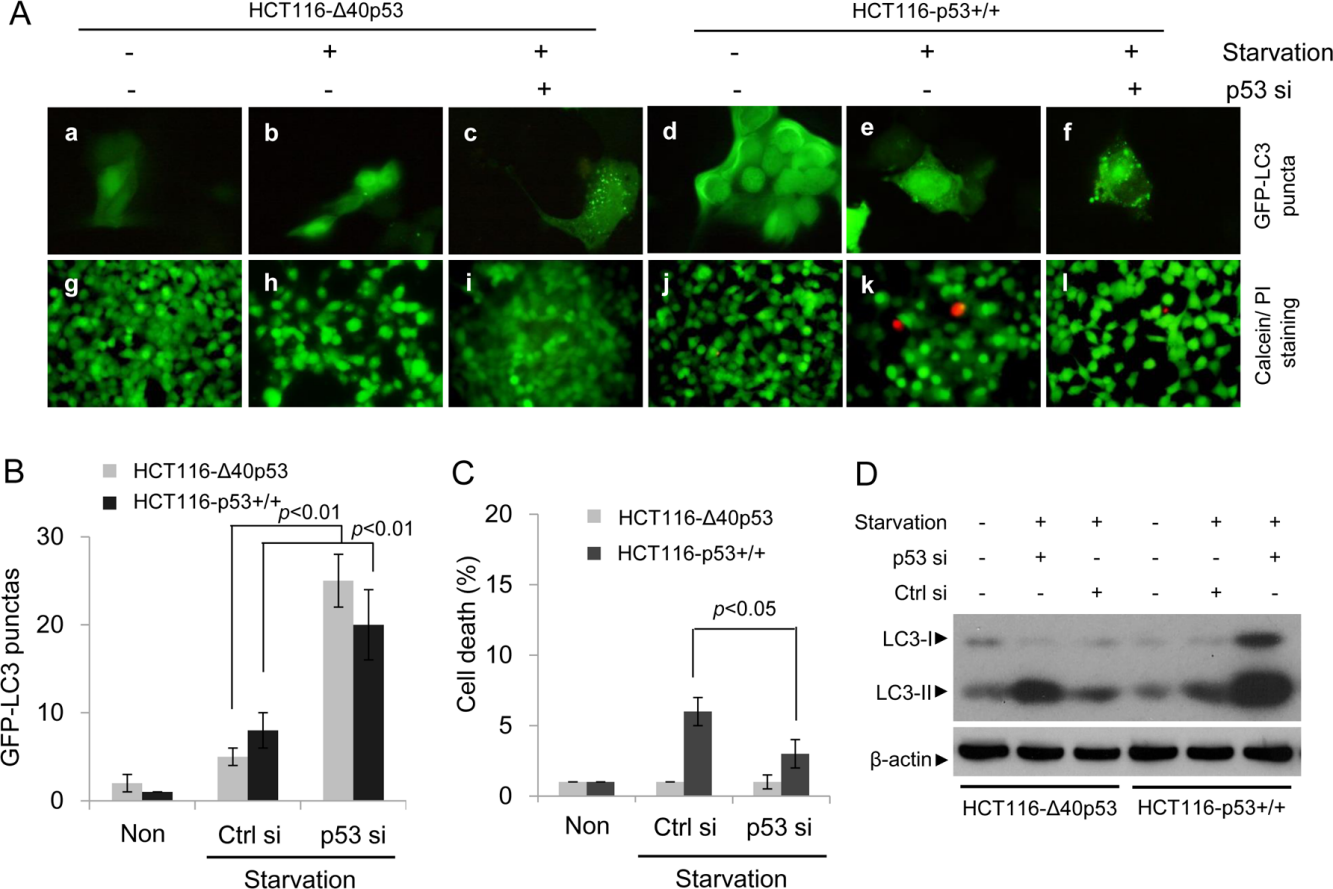
Δ40p53 inhibits starvation-induced autophagy in HCT116-Δ40p53 cells HCT116-p53+/+ and HCT116-Δ40p53 cells were transfected with a plasmid encoding GFP-LC3 and p53-directed siRNA (p53 si)/control siRNA (Ctrl si) for 24 hours and were then treated by starvation for 48 hours. Non: non-treatment. (**A**) Immunofluorescence detection of GFP-LC3 puncta (upper panel) and Calcein-AM/PI assay detection of cell death (lower panel). (**B, C**) Quantification of the number of GFP-LC3 puncta (**B**) and cell death (C) of (A). Data are presented as the mean ± SEM in three independent experiments. (**D**) Immunoblot detection using the indicated antibodies.

**Figure 3:**
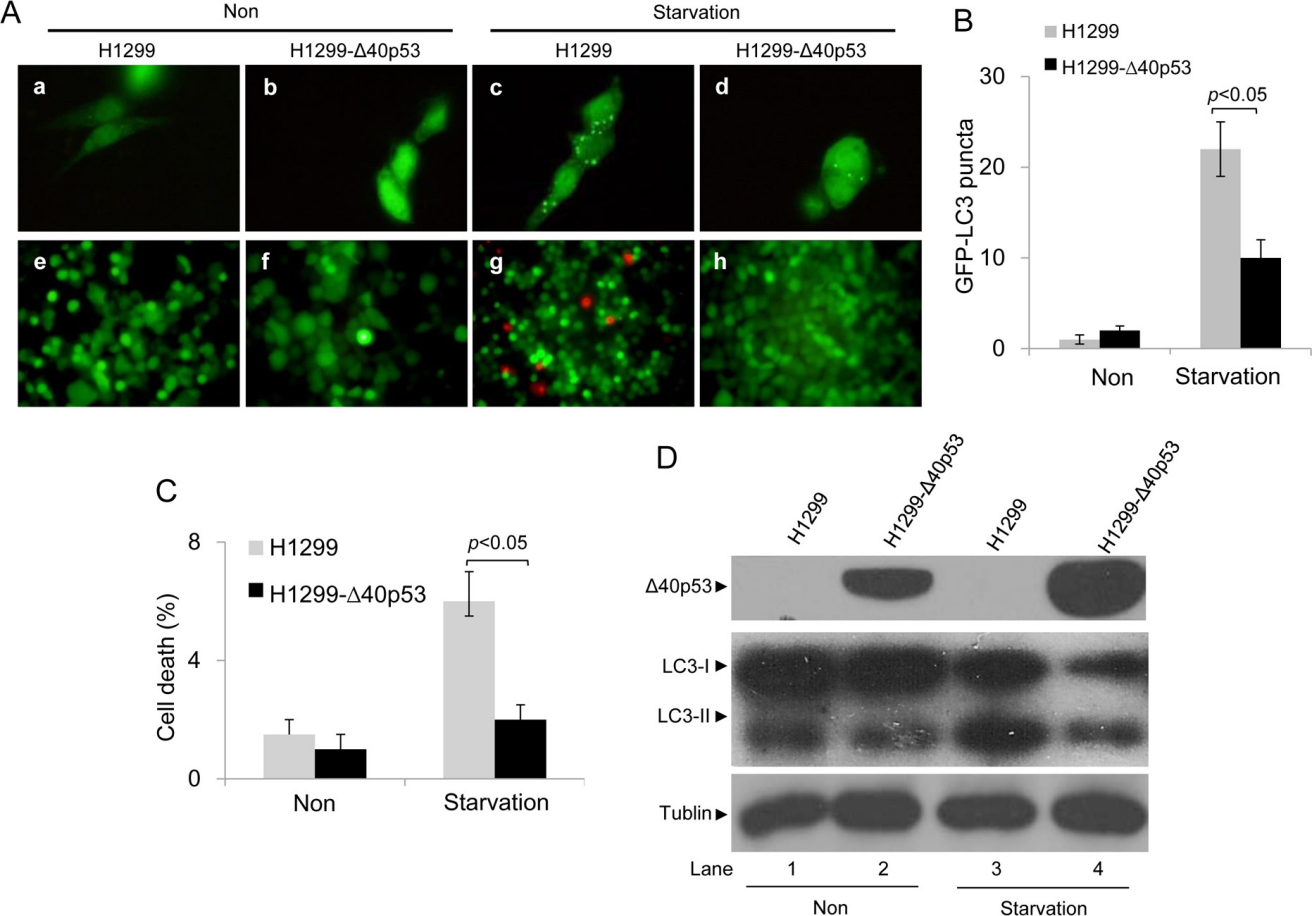
Δ40p53 inhibits starvation-induced autophagy in H1299-Δ40p53 cells H1299 (p53-free) and H1299-Δ40p53 cells were transfected with a plasmid encoding GFP-LC3 for 24 hours and were then treated by starvation for 48 hours. Non: non-treatment. (**A**) Immunofluorescence detection of GFP-LC3 puncta (upper panel) and Calcein-AM/PI assay detection of cell death (lower panel). (B, C) Quantification of the number of GFP-LC3 puncta (**B**) and cell death (C) of (A). Data are presented as the mean ± SEM in three independent experiments. (**D**) Immunoblot detection using the indicated antibodies.

HCT116-p53+/+ and HCT116-Δ40p53 cells were transfected with a plasmid encoding GFP-LC3 and were then treated with serum-free media, causing starvation. An immunofluorescence assay showed that starvation induced the formation of GFP-LC3 puncta in both cell lines (Figure 2A, 2B). When the expression of wtp53 and Δ40p53 were knocked down via p53-directed siRNA, the number of starvation-induced GFP-LC3 puncta was significantly higher than that observed with control siRNA treatment (Figure 2A, 2B). An immunoblot assay also showed that starvation increased the LC3-II/LC3-I ratio and that knockdown of wtp53 and Δ40p53 significantly increased this ratio in HCT116-p53+/+ and HCT116-Δ40p53 cells, respectively (Figure 2D). We also produced the cell line H1299-Δ40p53, which has stable expression of exogenous Δ40p53, by using the cell line H1299 (p53-free). H1299 and H1299-Δ40p53 were transfected with the GFP-LC3 plasmid and were then treated with serum-free media, causing starvation. Immunofluorescence and immunoblot assays showed that starvation increased GFP-LC3 puncta formation and the LC3-II/LC3-I ratio and that knockdown of wtp53 and Δ40p53 further enhanced GFP-LC3 punctum formation and the LC3-II/LC3-I ratio (Figure 3A, 3B, 3D). Thus, both Δ40p53 and wtp53 are inhibitors of starvation-induced autophagy.

We also detected the function of Δ40p53 in the induction of cell death using Calcein/PI staining. In HCT116-Δ40p53 and H1299-Δ40p53 cells, starvation and knockdown of Δ40p53 had no effect on the level of PI+ (dead) cells (Figures 2A, 2C, 3A, 3C). However, starvation increased the proportion of PI+ (dead) cells in HCT116-p53+/+ cell populations, and knockdown of wtp53 down-regulated starvation-induced cell death (Figure 2A, 2C). Starvation also increased the proportion of PI+ (dead) cells in H1299 cell populations but not in H1299-Δ40p53 cell populations, suggesting that Δ40p53 is an inhibitor of starvation-induced cell death (Figure 3A, 3C). Thus, unlike wtp53, Δ40p53 cannot induce cell death in cells treated by starvation.

### Like wtp53, the 3′-5′ exonuclease activity of Δ40p53 can also destroy double-stranded RNA

**Figure 4:**
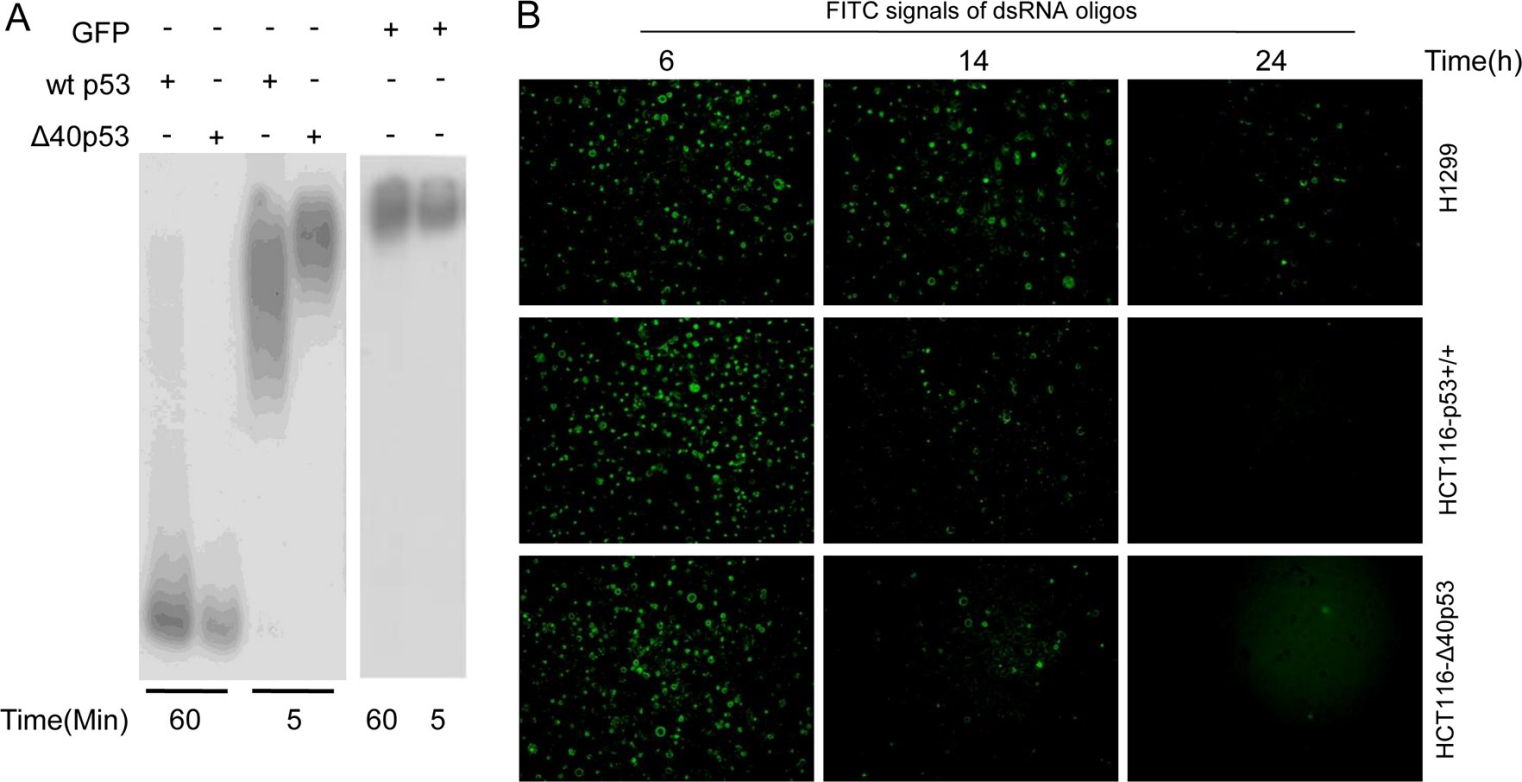
Δ40P53 can cleave double-stranded DNA (dsRNA) by its 3′-5′ exonuclease activity (**A**) p32-labeled double-stranded DNA was cultured with recombinant Δ40p53/wtp53 and GFP for 5 and 60 min. Autoradiography was used to detect the 3′-5′ exonuclease activity of recombinant Δ40p53 and wtp53. (**B**) FITC-labeled dsRNA oligos were transfected into H1299 (p53-free), HCT116-p53+/+ and HCT116-Δ40P53 cells. The detection of the number of FITC signals from dsRNA oligos in the three cell lines was measured to reflect the erasure of the FITC signal over 24 hours.

Previous studies have shown that cytoplasmic wtp53 inhibits autophagy by degrading double-stranded RNA (dsRNA) owing to its 3′-5′ exonuclease activity [2, 14]. Here, we determined that α-P^32^-dG-labeled PCR products were degraded by wtp53 and Δ40p53 when the PCR products and wtp53/Δ40p53 were co-cultured *in vitro* for 60 min (Figure 4A). Furthermore, FITC-labeled dsRNA oligos were transfected into H1299, HCT116-p53+/+ and HCT116-Δ40p53 cells, and then the time until the erasure of FITC fluorescence was detected. These immunofluorescence assays showed that the FITC signal was almost undetectable at 24 hours in the HCT116-p53+/+ and HCT116-Δ40p53 cells but was still easily detectable in H1299 cells at this time point (Figure 4B). These data suggest that Δ40p53 has 3′-5′ exonuclease activity as does wtp53, which results in autophagy inhibition.

### Cytoplasmic Δ40p53 inhibits starvation-induced autophagy by inactivation of the PKR/elF2α pathway

**Figure 5:**
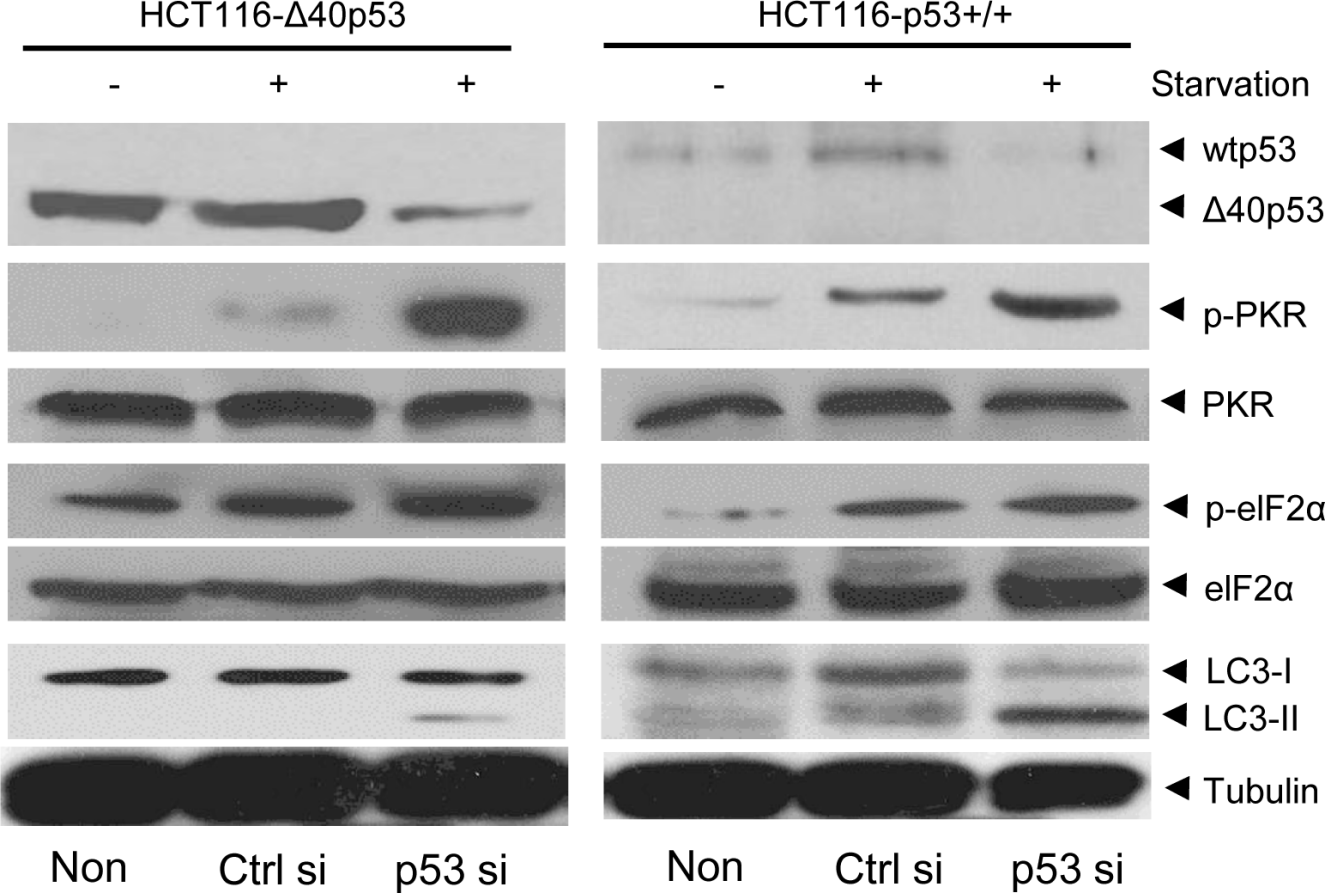
Δ40p53 inhibits the phosphorylation of PKR and elF2α HCT116-p53+/+ and HCT116-Δ40p53 cells were transfected with p53-directed siRNA (p53 si)/control siRNA (Ctrl si) for 24 hours and were then treated by starvation for 48 hours. Non: non-treatment. Immunoblot detection with the indicated antibodies.

dsRNA can activate the PKR/elF2α pathway by inducing the phosphorylation of PKR and elF2α, which contributes to induction of expression of some autophagy-related genes and subsequently induces autophagy [15, 16]. Here, starvation induced the phosphorylation of PKR and elF2α in HCT116-p53+/+ and HCT116-Δ40p53 cells, but knockdown of wtp53 and Δ40p53 via p53-directed siRNA contributed to higher levels of p-PKR and p-elF2α than did control siRNA treatment (Figure 5). These data suggest that, like wtp53, Δ40p53 can inhibit, at least partly, autophagy by inhibiting the phosphorylation of PKR/elF2α through its 3′-5′ exonuclease activity.

### Nuclear Δ40p53 cannot induce autophagy by inducing DRAM expression and inhibits the transactivation ability of wtp53

**Figure 6:**
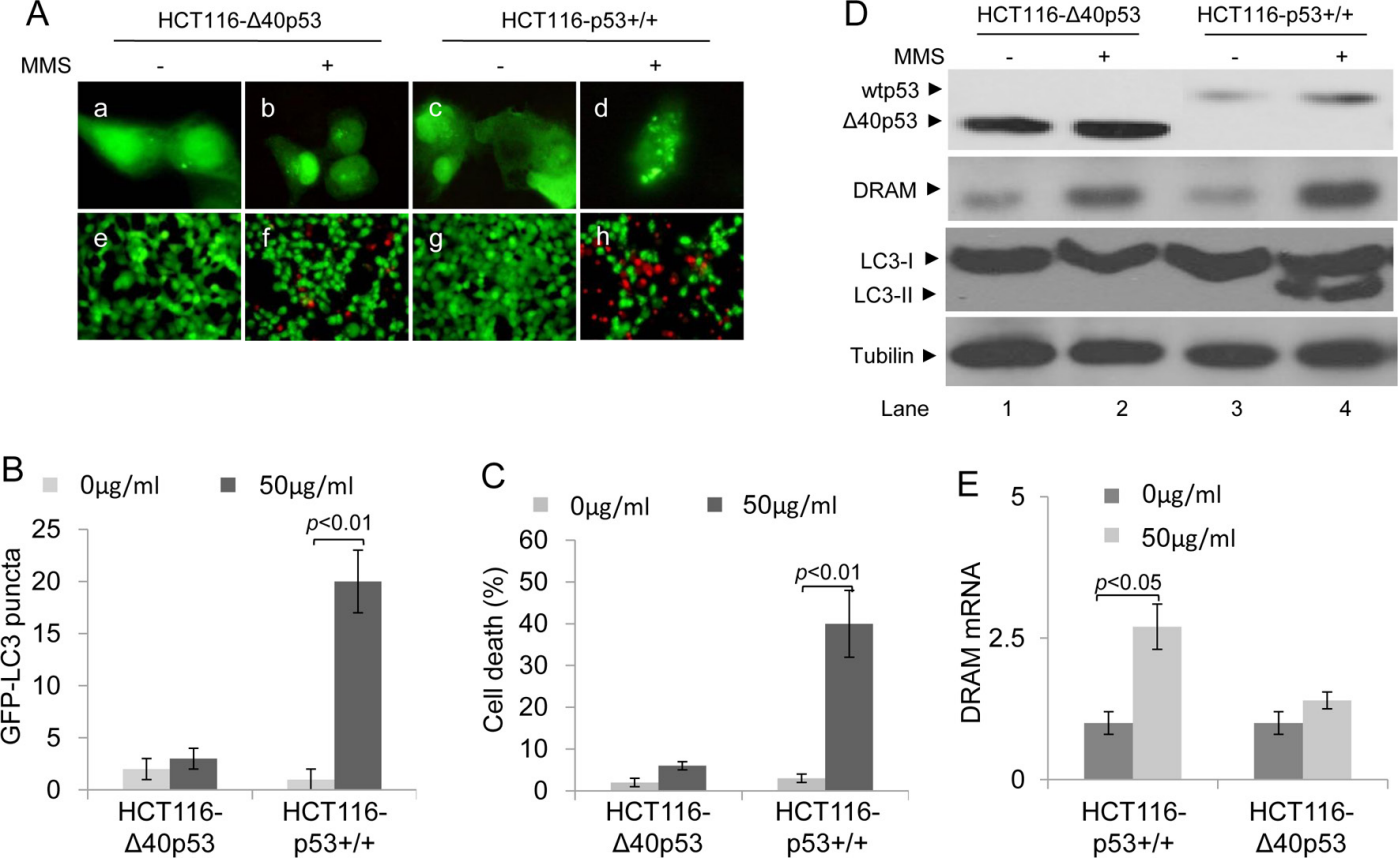
Δ40p53 cannot induce autophagy and cell death in HCT116-Δ40p53 cells in response to MMS treatment HCT116-p53+/+ and HCT116-Δ40p53 cells were transfected with a plasmid encoding GFP-LC3 for 24 hours and then treated with 50 μg/ml MMS for 48 hours. (**A**) Immunofluorescence detection of GFP-LC3 puncta (upper panel) and Calcein-AM/PI assay detection of cell death (lower panel). (B, C) Quantification of the number of GFP-LC3 puncta (**B**) and cell death (C) of (A). Data are presented as the mean ± SEM in three independent experiments. (**D**) Immunoblot detection using the indicated antibodies. (**E**) qRT-PCR detection of DRAM mRNA.

Nuclear wtp53 can induce autophagic apoptosis, which contributes to cell death, by promoting the expression of wtp53 target genes: e.g., DRAM [5]. Our previous data have shown that most of the Δ40p53 molecules translocate to the nucleus in response to MMS-induced DNA damage. In HCT116-p53+/+ cells treated with MMS, a significant increase in GFP-LC3 puncta (to 23 ~ 30 puncta/cell) and PI+ (dead) cells was detected; however, in HCT116-Δ40p53 cells treated with MMS, no significant increase in GFP-LC3 puncta (to 1 ~ 4 puncta/cell) and PI+ (dead) cells was detected (Figure 6A, 6B, 6C). An immunoblot assay also showed that MMS treatment increased the LC3-II/LC3-I ratio only in the presence of wtp53 but not Δ40p53 (Figure 6D). These data suggest that whereas wtp53 induces autophagy and cell death in response to MMS treatment, Δ40p53 does not. An immunoblot assay showed that the expression of DRAM increased by 3-fold and by 10-fold in the HCT116-Δ40p53 and HCT116-p53+/+ cells, respectively (Figure 6D). DRAM mRNA significantly increased in HCT116-p53+/+ cells, but not HCT116-Δ40p53 cells, treated with MMS (Figure 6E). Thus, our data suggest that although Δ40p53 translocates to the nucleus in response to MMS-induced DNA damage, nuclear Δ40p53 cannot induce DRAM expression; therefore, it cannot induce cell death by promoting DRAM-induced autophagic apoptosis.

**Figure 7:**
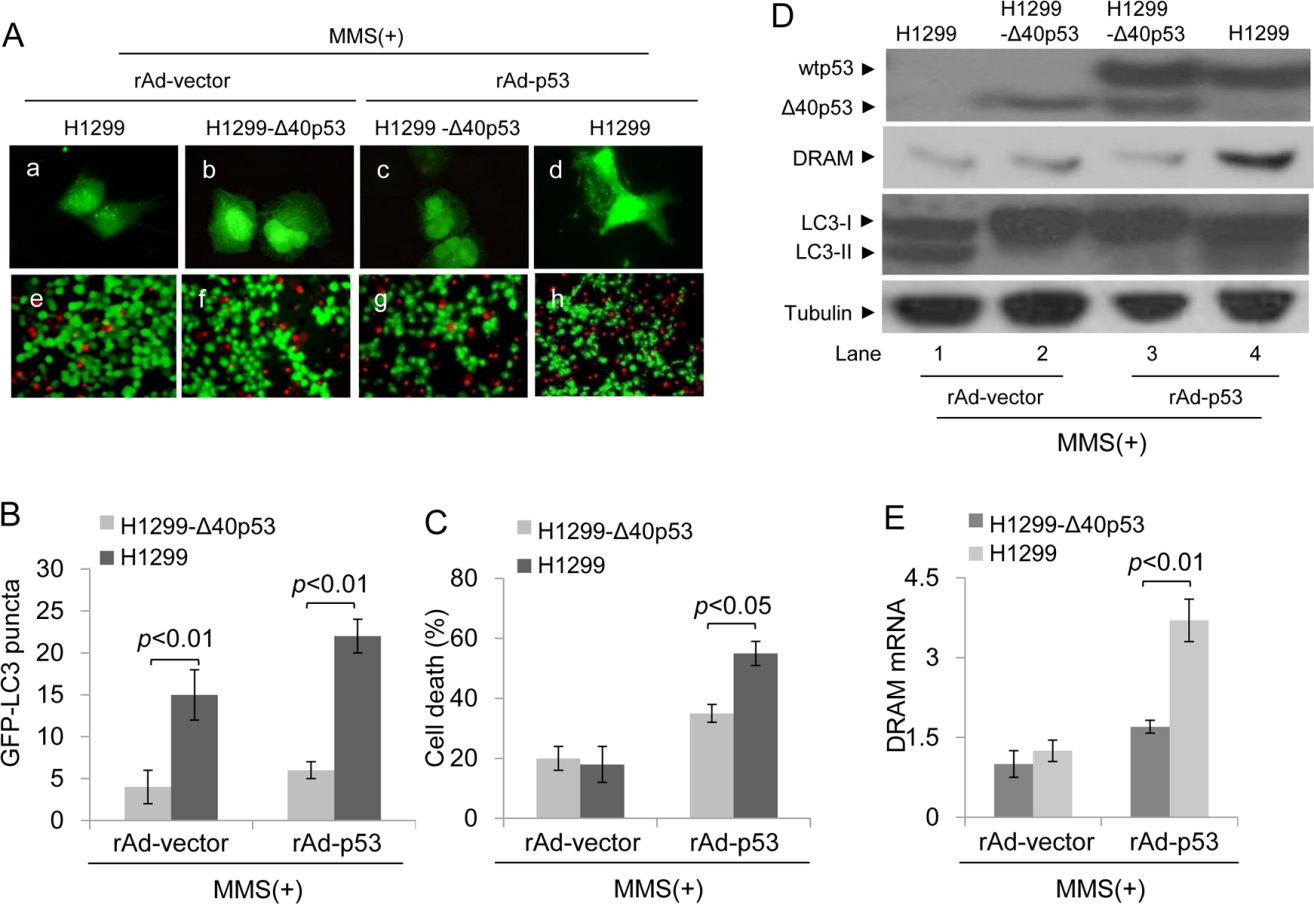
Δ40p53 cannot induce autophagy and cell death in H1299-Δ40p53 cells in response to MMS treatment H1299 (p53-free) and H1299-Δ40p53 cells were transfected with a plasmid encoding GFP-LC3 and infected with recombinant adenovirus p53 (rAd-p53)/vector (rAd-vector) for 24 hours and then treated with 50 μg/ml MMS for 48 hours. (**A**) Immunofluorescence detection of GFP-LC3 puncta (upper panel) and Calcein-AM/PI assay detection of cell death (lower panel). (B, C) Quantification of the number of GFP-LC3 puncta (**B**) and cell death (C) of (A). Data are presented as the mean ± SEM in three independent experiments. (**D**) Immunoblot detection using the indicated antibodies. (**E**) qRT-PCR detection of DRAM mRNA.

Next, we assessed whether Δ40p53 affects the function of wtp53 in inducing cell death when Δ40p53 and wtp53 co-translocate to the nucleus in response to MMS-induced DNA damage. H1299 and H1299-Δ40p53 cells were infected by recombinant adenovirus carrying wtp53 (rAd-p53) or vector only (rAd-vector) and were then treated with MMS. An immunofluorescence assay showed that MMS or MMS plus rAd-p53 infection induced more GFP-LC3 puncta in H1299 cells than in H1299-Δ40p53 cells (Figure 7A, 7B). These data suggest that Δ40p53 inhibits DNA damage-induced and wtp53-induced autophagy. The Calcein/PI staining assay showed that the proportion of PI+ (dead) cells was significantly lower in H1299-Δ40p53 cell populations than in H1299 cell populations in response to MMS plus rAd-p53 infection, suggesting that Δ40p53 inhibits the function of wtp53 in inducing cell death (Figure 7A, 7C). An immunoblot assay showed that DRAM only increased in H1299 cells treated with MMS plus rAd-p53 infection, and the LC3-II/LC3-I ratio significantly increased in H1299 cells treated with MMS alone or MMS plus rAd-p53 infection (Figure 7D). The qRT-PCR assay further showed that the level of DRAM mRNA in H1299-Δ40p53 cells was significantly lower than that of H1299 cells in response to MMS plus rAd-p53 infection, suggesting that Δ40p53 inhibits the transactivation activity of wtp53 in inducing DRAM expression (Figure 7E).

## DISCUSSION

Δ40p53 was first discovered in 1985, but its functions have still not been clearly elucidated. In this study, we assessed whether Δ40p53 affects the function of wtp53 and individually regulates autophagy and cell death. We found that in the cytoplasm, both Δ40p53 and wtp53 use their 3′-5′ exonuclease capacity to inhibit autophagy. To our knowledge, this is the first study to report that Δ40p53 has 3′-5′ exonuclease capacity, which contributes to its role in inhibiting autophagy. Autophagy is an intracellular degradation process that is essential for the survival of eukaryotic cells and mammals, as well as for cell homeostasis, development, stemness of normal cells/cancer cells, tumorigenesis and infection [17]. However, autophagy is also regarded as type II programmed cell death (also termed autophagic apoptosis) [18]. In addition, many studies suggest that autophagy is a potential therapeutic target for improving human diseases and conditions, such as cancer, diabetes, neurodegeneration and infection [17]. Thus, cytoplasmic Δ40p53 might affect the above-mentioned physiological functions and disease development by regulating autophagy development. Although our study showed that the 3′-5′ exonuclease capacity of Δ40p53 contributes to its autophagy-inhibiting function, we believe that there may be additional mechanisms by which Δ40p53 plays a role in inhibiting autophagy.

In addition to inhibition of autophagy, it is reasonable to believe that Δ40p53 has many other functions given its 3′-5′ exonuclease capacity. These functions of Δ40p53 may be able to be predicted by considering the 3′-5′ exonuclease capacity of wtp53. The dsRNase activity of wtp53 activates PKR and eIF2α, which inhibits viral protein synthesis and viral replication, suppresses the growth of tumor cells and increases p53 transcription activity [19]. Thus, Δ40p53 is likely to also have these functions, including tumor growth inhibition and viral infection suppression in some special conditions, suggesting that Δ40p53 may be a good target for treating tumors and viral infection.

Here, we identified that another important function of Δ40p53 is inhibiting the transactivation capacity of wtp53 in the nucleus. The phenomenon that both Δ40p53 and wtp53 can be co-localized in the nucleus has been reported [19]. Here, we also observed co-localization of Δ40p53 and wtp53 in the nucleus of cells exposed to MMS-induced DNA damage. However, the dominant effects of nuclear Δ40p53 on wtp53 functions are contradictory. For example, Δ40p53 formed nuclear hetero-tetramers with activated wtp53 and increased promoter occupancy at both the PIDD and p21 genes [20]. However, other studies have shown that Δ40p53 cannot induce apoptosis but negatively regulates the transactivation capacity of wtp53 [9, 21]. Our data show that nuclear Δ40p53 inhibits the transactivation capacity of wtp53 and reduces wtp53-induced cell death. The exact mechanism by which Δ40p53 inhibits p53 function needs to be further studied. However, a previous study suggests that the presence of Δ40p53 can result in translocation of wtp53 from the nucleus to the cytoplasm when cells are stimulated [9]. This suggests that Δ40p53 might, at least in part, promote the cytoplasmic translocation of wtp53 from the nucleus, resulting in the inhibition of p53-induced cell death.

In summary, our data uncover new roles of Δ40p53 in the regulation of autophagy and cell death in cells. Our findings suggest that Δ40p53 might be a good target for developing treatments for tumors and viral infections.

## MATERIALS AND METHODS

### Cell culture, protein expression and reagents

Cells were cultured in DMEM containing 10% heat-treated fetal bovine serum (FBS), L-glutamine (290 μg/ml), penicillin (100 U/ml) and streptomycin (100 U/ml) and were maintained in logarithmic growth at 37°C in a 5% CO_2_ incubator. The cell line HCT116-p53+/+ was a gift from Dr. Charles Lopez (OHSU, Portland, OR) [22]. Two cell lines (HCT116-Δ40p53 and H1299-Δ40p53) expressing only Δ40p53 but not wtp53 were made by He Yuan Bio-Inc (Shanghai, China) from HCT116-p53+/+ and H1299 cells (p53-free). For adenovirus infection, a recombinant adenovirus encoding p53 (rAd-p53) and an empty vector (rAd-vector) were made using the AdEasy adenoviral vector system (Stratagene).

### *In vitro* 3′-5′ exonuclease activity assay

Recombinant wtp53, Δ40p53, and green fluorescent protein (GFP) were generated by cloning the cDNAs into a His-tagged expression vector (Invitrogen, Carlsbad, CA) using standard techniques. Proteins were purified on Ni-NTA agarose and stored at −80°C after quantification of proteins using standard techniques. *In vitro* 3′-5′ exonuclease activity was detected by using the α-^32^p-dG-PCR product. Briefly, 1 μg of wtp53, Δ40p53 or GFP fusion protein was incubated with α-^32^p-dG-PCR product in a 20-μl volume at 32°C for 5 and 60 min. Reactions were terminated with loading buffer (90% formamide, 0.002% bromophenol blue, 0.002% xylene cyanol), and solutions were heated to 80°C for 2 min, with the proteins subsequently resolved on a denaturing 6% polyacrylamide / 7 mmol/L urea gel, which was imaged by autoradiography.

### Calcein-AM/PI assay

Cell death was assessed by the Calcein-AM/PI assay. The detailed method was described previously [23]. Briefly, cells were incubated with 1 μg/mL calcein-AM for 30 min; 10 μg/mL PI was then added for 15 min in medium at 37°C. Cell mortality is expressed as the ratio of PI^+^ cells/ all cells.

### Immunoblotting

The anti-p53 antibodies 1801 and DO1 were purchased from NewMarker (USA). The antibodies for detecting MDM2, tubulin, p-PKR, PKR, eIF2, p-elF2 were purchased from Santa Cruz (USA). The anti-LC3 polyclonal antibody was purchased from Abcam (USA). The cell preparation and standard immunoblotting method were described previously [24].

### qRT-PCR assay

An RNeasy Mini Kit (Qiagen, Hilden, Germany) was used to isolate total RNA. Reverse transcription was accomplished using a SuperScript III First-strand Synthesis System for RT-PCR (Invitrogen, Carlsbad, CA) to synthesize first-strand cDNA. SYBR green was used to detect the dsDNA product during qRT-PCR. The mRNA value was normalized to the housekeeping gene β-actin [24]. Specific primer sequences used for real time PCR were as follows: for DRAM, 5′-TCAAATATCACCATTGATTTCTGT-3′ (forward) and 5′-GCCACATACGGATGGTCATCTCTG-3′ (reverse) (the sequences of DRAM primers were from reference 23); for β-actin, 5′-GCCCTGAGGCACTCTTCCA-3′ (forward) and 5′-CGGATGTCCACGTCACACTT-3′ (reverse). To confirm the presence of Δ40p53 and p53 mRNAs, two primer pairs were designed. Both primer pairs possess the same reverse primer (p53-AS12, 5′-GGAAACC GTAGCTGCCCTGGTAGG-3′). One forward primer (p53SE2, 5′-GAGCCGCAGTCAGATCCTAGCGTC-3′) was designed to amplify from exon 2; the other forward primer (p53SE4, 5′-CCGGACGATATTGAAC AATGGTTC-3′) amplifies from exon 4. Thus, the primer pair p53SE2/p53-AS12 cannot detect Δ40p53 mRNA but only wtp53 mRNA. The primer pair p53SE4/p53-AS12 can detect both wtp53 and Δ40p53 mRNA.

### Immunofluorescence staining and quantification of GFP-LC3 puncta

Cells (1 × 10^5^) were seeded on 6-well plates and were then treated with 50 μg/ml MMS for 15 hours. Cells were fixed with 1% formaldehyde and permeabilized with PBS containing 0.1% Triton X-100. Cells were blocked for one hour and then incubated overnight at 4°C with anti-p53 monoclonal primary antibody (1801) diluted with blocking buffer. After washing with PBS, cells were incubated with CY3-conjugated anti-IgG (Jackson Immunochemicals) diluted with blocking buffer. The number of GFP-LC3 puncta was quantified in cells showing accumulation of GFP-LC3 in dots or vacuoles (total of a minimum of 100 cells per preparation in three independent experiments). Cells presenting a mostly diffuse distribution of GFP-LC3 in the cytoplasm and nucleus were considered non-autophagic.

### Statistical analysis

Student's t-distribution probability density function was used for calculation of the *p*-value.
